# Decision support for evidence-based integration of disease control: A proof of concept for malaria and schistosomiasis

**DOI:** 10.1371/journal.pntd.0006328

**Published:** 2018-04-12

**Authors:** Claire J. Standley, Ellie Graeden, Justin Kerr, Erin M. Sorrell, Rebecca Katz

**Affiliations:** 1 Center for Global Health Science and Security, Georgetown University, Washington, DC, United States of America; 2 Talus Analytics, LLC, Lyons, CO, United States of America; University of Washington, UNITED STATES

## Abstract

Managing infectious disease requires rapid and effective response to support decision making. The decisions are complex and require understanding of the diseases, disease intervention and control measures, and the disease-relevant characteristics of the local community. Though disease modeling frameworks have been developed to address these questions, the complexity of current models presents a significant barrier to community-level decision makers in using the outputs of the most scientifically robust methods to support pragmatic decisions about implementing a public health response effort, even for endemic diseases with which they are already familiar. Here, we describe the development of an application available on the internet, including from mobile devices, with a simple user interface, to support on-the-ground decision-making for integrating disease control programs, given local conditions and practical constraints. The model upon which the tool is built provides predictive analysis for the effectiveness of integration of schistosomiasis and malaria control, two diseases with extensive geographical and epidemiological overlap, and which result in significant morbidity and mortality in affected regions. Working with data from countries across sub-Saharan Africa and the Middle East, we present a proof-of-principle method and corresponding prototype tool to provide guidance on how to optimize integration of vertical disease control programs. This method and tool demonstrate significant progress in effectively translating the best available scientific models to support practical decision making on the ground with the potential to significantly increase the efficacy and cost-effectiveness of disease control.

**Author summary:**

Designing and implementing effective programs for infectious disease control requires complex decision-making, informed by an understanding of the diseases, the types of disease interventions and control measures available, and the disease-relevant characteristics of the local community. Though disease modeling frameworks have been developed to address these questions and support decision-making, the complexity of current models presents a significant barrier to on-the-ground end users. The picture is further complicated when considering approaches for integration of different disease control programs, where co-infection dynamics, treatment interactions, and other variables must also be taken into account. Here, we describe the development of an application available on the internet with a simple user interface, to support on-the-ground decision-making for integrating disease control, given local conditions and practical constraints. The model upon which the tool is built provides predictive analysis for the effectiveness of integration of schistosomiasis and malaria control, two diseases with extensive geographical and epidemiological overlap. This proof-of-concept method and tool demonstrate significant progress in effectively translating the best available scientific models to support pragmatic decision-making on the ground, with the potential to significantly increase the impact and cost-effectiveness of disease control.

## Introduction

The concept of integrated control of neglected tropical diseases (NTDs) as a public policy was established more than a decade ago. The proposed policy highlighted the potential benefits of “rapid-impact interventions”, particularly bundled combinations of drugs to be used for mass drug administration (MDA) campaigns at school or the community level[1]. Large scale implementation of integrated, or at least coordinated, programs began soon after: in 2006, the United States Agency for International Development (USAID) launched an NTD Control Program, which included an explicit focus on coordination of control interventions in its twelve target countries[2]. Since then, a number of countries have begun to implement control programs that integrate two or more neglected tropical diseases, largely focusing on coordinated MDA, but also occasionally incorporating other interventions[3]. While primarily focused on integration of NTDs, from the beginning there have been calls to “piggy-back” NTD control through integration with the “Big Three” of HIV/AIDS, tuberculosis (TB) and/or malaria given the substantially larger resources allocated to these three major diseases and significant existing infrastructure for implementation of control measures, including drug delivery, community outreach, and advocacy[4,5].

The integration of NTDs, either alone or with other health and disease programs, including the Big Three, is supported by, first, the extensive geographical overlap of these diseases, resulting in high prevalence of co-infection. Secondly, known immunological and pathological dynamics in co-infected individuals can lead to synergistic impacts and changes in susceptibility to other infections, including HIV and malaria. Thirdly, integrated programs can benefit from significant financial savings generated by greater efficiency of intervention delivery, for example through fewer and more stream-lined MDA campaigns. The past ten-plus years of integrated control have provided *a posteriori* evidence of efficacy, particularly in terms of numbers of drug treatments delivered, national coverage rates, and, in some cases, through cost effectiveness estimates. While there are concerns that monitoring and evaluation efforts have not always kept pace with integration initiatives, that observed cost-savings are variable and vulnerable to opportunity costs, and that context-specific factors may influence the execution and impact of integrated control[6–8], cumulative evidence suggests that, at least in some cases, integrated control measures could significantly improve the global impact of co-infection and total disease load.

The effectiveness of integrated disease control programs depends on the specific co-endemic diseases, infection patterns, and population structure in the local community. However, those making public health decisions on the ground rarely have access to decision support tools that translate the outputs of the best available models into practical decisions for implementing public health efforts in their communities. To address this gap, we have developed an evidence-based framework to support *a priori* design, implementation, and monitoring of integrated disease control programs. This approach applies epidemiological and immunological data with context-specific demographic information to predict whether integration would be beneficial in a particular setting. This approach is specifically designed to facilitate the transfer of information usually restricted to academic literature into the hands of end-users and decision-makers on the ground.

Schistosomiasis and malaria are ideal targets for testing the validity of this type of evidence-based *a priori* framework for decision-making. The two diseases are co-endemic throughout much of sub-Saharan Africa, as well as certain regions of South America, the Middle East, and Southeast Asia, and have similar demographic impacts, with children experiencing the highest burden of morbidity. The two diseases are known to cause significant rates of co-infection at the individual level[9–12] and are known to have complex immunological and pathological interactions.[11,13,14] In addition, individuals who are heavily infected with *Schistosoma mansoni* may have increased malaria susceptibility[13], suggesting that integration of control programs for both diseases could yield significant additive benefits. Finally, while schistosomiasis is characterized as an NTD, malaria is one of the well-funded Big Three, and thus efforts to integrate these two diseases can provide insight into the opportunities for leveraging well-resourced disease control programs to assist programs for diseases with limited funding.

Here, we present a decision-support tool, focused on schistosomiasis and malaria at the individual and population levels, designed to support disease control programs in determining not only whether there is a predicted benefit to integration in their setting, but also how best to implement integration, based on the local context of existing and planned interventions.

## Methods

To support local public health officials and policy makers evaluate whether an integrated control strategy for malaria and schistosomiasis would be beneficial for their community, we adapted disease modeling approaches[15,16] for non-expert users and designed a web-based decision support tool. The decision support tool includes parameters to test the beneficial effects of insecticide-treated bed nets (ITNs), indoor residual insecticide spraying (IRS), and mass drug administration of praziquantel (PZQ) to treat schistosomiasis infection. Model parameters for disease transmission rates, access to malaria treatment, and efficacy of disease control interventions were based on those identified in the literature for regions with high co-infection rates of endemic schistosomiasis and malaria. The remaining model parameters were constrained to those data that community leaders and public health practitioners can supply about the current disease control programs and basic information about malaria transmission in their region, including seasonality of malaria transmission and schistosomiasis prevalence. The user configures model input parameters via a web-based graphical user interface (GUI) and the model is run twice: once simulating integrated treatment and once simulating non-integrated treatment. By comparing model runs for the current disease control programs with an integrated strategy, the decision support tool provides users with a recommendation of whether an integrated control program is beneficial based on quantitative estimates of malaria prevalence calculated in the disease model.

### Model

#### Modeling approach

**Fig 1:**
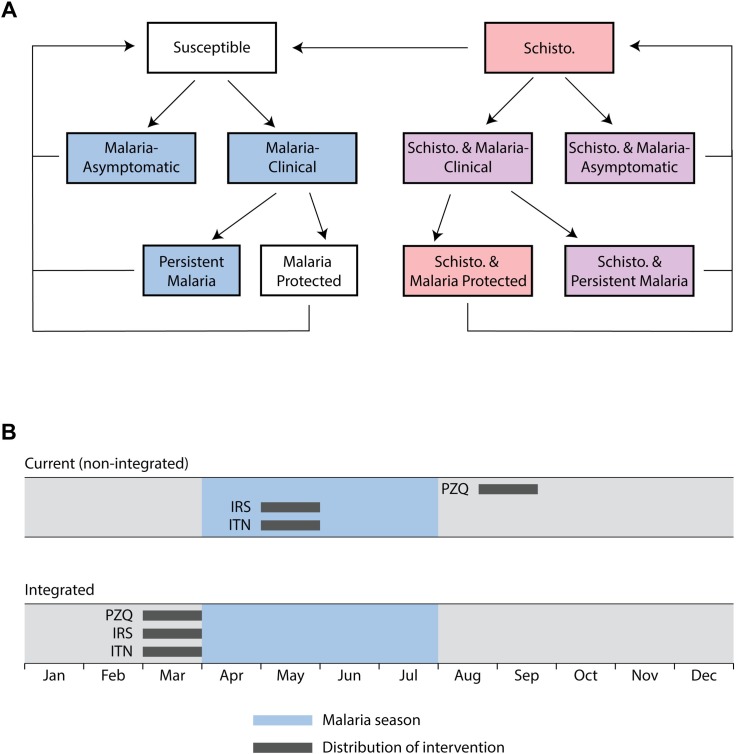
Schematic framework for malaria schistosomiasis co-infection model to evaluation of integrated control programs. (A) Individuals enter the model either disease-free (left) or infected with schistosomiasis (right). Diagram shows transition between disease states of the modeled population (arrows). Praziquantel MDA is assumed effective in curing schistosomiasis in all individuals with access to treatment. Blue = malaria, red = schistosomiasis (schisto.), and purple = co-infection. (B) The model performs two simulations for each user submission to the decision support tool. The current (non-integrated) simulation delivers control measures with timing specified by the user. The Integrated simulation aligns all interventions to occur at the same time. For non-seasonal transmission, interventions are aligned to all occur at the earliest intervention time specified by the user. For seasonal transmission, all control measures are delivered one month prior to the start of malaria season. Malaria prevalence estimates for these two simulations are compared to evaluate the potential benefits of integration.

As described in additional detail in the following sections, population-level prevalence reported by the decision support tool is based on an underlying disease model that tracks schistosomiasis and malaria disease states for each individual as he/she passes through malaria infected, schistosomiasis, and co-infected states over time (Fig 1A). Each simulation begins with individuals entering the model either disease-free (Fig 1A, left) or schistosomiasis-infected (Fig 1A, right), with infections assigned to individuals at random based on the population-level prevalence specified by the user. Malaria infections are introduced and the model is equilibrated with a “burn in” period of at least 730 days to approximate the user-specified transmission level before interventions are tested. After the burn in period, control measures directly modulate probability of malaria infection for each individual. Adapting malaria disease parameters previously published, 72% of individuals develop clinical malaria upon infection [16] and seek treatment (Fig 1A, Malaria-clinical). Among clinical cases, individuals with access to treatment receive artemisinin-based combination therapy (ACT) treatment that cures and temporarily protects from malaria infection; we assume maximal treatment access for 76% of cases, based on the best available data for Uganda.[17] Asymptomatic and untreated clinical malaria (persistent malaria) cases are cleared without treatment, returning individuals to susceptible or schistosomiasis-infected states after 360 days.[15]

Central to our decision support method is comparison of predicted prevalence under the current control program, presumed to be non-integrated, with an integration of control measures for both diseases. To compare these conditions and provide users with an overall recommendation regarding the potential benefit of integrated control on malaria prevalence, two model runs are performed under each condition (Fig 1B). The current (non-integrated) simulation applies malaria and schistosomiasis control measures using the current timing of intervention strategies, as defined by the user. Fig 1B (upper timeline) shows an example community where the current distribution of malaria control measures, IRS and ITN, occurs in May during peak malaria transmission and PZQ is administered in September. This simulation predicts prevalence under the “status quo” and provides the reference to which the integrated control program simulation is compared. The integrated simulation (Fig 1B, lower timeline) models the simultaneous distribution of control measures for both schistosomiasis and malaria and provides results for predicted disease prevalence for a hypothetical integrated intervention program to compare with the current, non-integrated program.

The timing of control measure distribution for the integrated intervention program depends on whether malaria transmission is seasonal or continuous throughout the year (non-seasonal). If malaria in the community is non-seasonal, interventions are aligned at the first intervention time specified by the user. For regions with a seasonal increase in malaria transmission, as in Fig 1B, interventions are modeled as executed one month prior to the seasonal onset. Administering PZQ prior to malaria season, as shown for the integrated control program, is predicted to provide the most benefit by reducing malaria susceptibility for schistosomiasis-infected individuals prior to peak transmission months. In addition, this timing is supported by previous studies that have demonstrated maximal benefits for IRS when spraying is completed shortly before the onset of malaria season[18], as in the integrated approach employed in this model.

Table 1 summarizes key time points for malaria transmission and the timing of interventions. Users specify months of increased seasonal malaria transmission, if any; a five-fold increase in malaria infection probability is implemented for each day of the selected months (see Malaria Infections section below). The burn-in period is longer ($N_{days}^{burn}$ plus the number of months that have passed since malaria season began) for seasonal transmission to ensure the model simulates control measures during a full malaria season after stabilization of baseline transmission. Non-integrated control measures are modeled with timing specified by the user. When defining integrated control, interventions are distributed one month before malaria season begins. For seasonal increases with multiple peaks in transmission, interventions are distributed one month before the first peak in the calendar year. In all cases, the model applies non-integrated control measures on the first day of the month selected by the user for that intervention (IRS, ITN, or PZQ).

**Table 1:** Key time points on the simulation timeline. The methods used to determine time points for both seasonal and continuous malaria transmission patterns.

Time point name	Seasonal	Continuous
Malaria season dates	Includes each day of each month selected by user for malaria season	N/A
PZQ distribution date: With integration	1 month before first day of the earliest calendar month in malaria season	First day of month input by user for earliest intervention
PZQ distribution date: Without integration	First day of month input by user for PZQ distribution	First day of month input by user for PZQ distribution
ITN distribution date	Same as described for PZQ distribution date above	
IRS distribution date		
Simulation start date	First day of distribution month for earliest intervention	
Simulation end date	Simulation start date plus *N*_*days*_ (365 days)	
Burn-in period start date	At least $N_{days}^{burn}$ (730 days) before simulation start date	
Burn-in period end date	Simulation start date	

The model uses a stochastic, object-oriented programming approach, written in the programming language Python. New disease infections (schistosomiasis and malaria), access to malaria treatment, and access to control measures (MDA, ITN, and IRS) are each assigned stochastically (at random) using either fixed or user-selected model parameters (e.g. for 80% ITN coverage, each individual has an 80% probability of receiving bed net protection), as described below.

#### Schistosomiasis infections

Upon initiation of the model, each person has a probability of being infected with schistosomiasis *p*_*s*_, where *p*_*s*_ is calculated directly from the user-supplied prevalence of schistosomiasis (e.g., 45% population prevalence corresponds to *p*_*s*_ = 0.45, see additional details in Table 2 below). The model assumes a constant schistosomiasis infection state for each individual; no additional schistosomiasis infections are modeled. This focused approach supports evaluation of integrated control programs specifically in the context of the indirect benefit of MDA in reducing malaria susceptibility. Importantly, tracking of schistosomiasis-infected individuals is used in the model to have an 85% increase in malaria susceptibility.[13,15] Schistosomiasis is “cured” in the model following treatment with PZQ, at which point malaria susceptibility returns to the baseline level.

**Table 2:** User-assigned demographic breakdown and schistosomiasis parameters.

**Population: Age distribution**			
Parameter name	Description	Default value	Possible values
Age range: under 5	Percent of population under 5 years old	20% [20]	Percentage value between 0 and 100
Age range: 5–15	Percent of population 5 to 15 years old	34% [20]	Percentage value between 0 and 100
Age range: 16+	Percent of population 16 or more years old	46% [20]	Percentage value between 0 and 100
**Schistosomiasis: Praziquantel (PZQ) Drug Administration**			
Parameter name	Description	Default value	Possible values
*p*_*s*_	Fraction of population initially infected with schistosomiasis	45% [21–24]	Percentage value between 0 and 100
Target % Coverage	Target percent coverage of PZQ distribution	80% [25]	30%, 50%, 80%, 100%
Age Range	Age ranges that receive PZQ	5–15 years old [19]	Any selection(s) from: under 5, 5–15, 16+
Current Distribution Month	Month PZQ is distributed (for non-integrated)	April	Any calendar month (e.g., “April”)

#### Malaria infections

Malaria transmission is defined by the annual entomological infection rate (AEIR) and adjusted for seasonal increases in transmission, the protective interventions (IRS and ITN), and increased susceptibility to malaria infection for schistosomiasis-infected individuals and these factors are combined in calculating the probability of a susceptible person contracting a new malaria infection (*p*_*m*_(*t*)). Changes in transmission are represented by varying AEIR, but mosquito vectors are not explicitly modeled. Specifically, the daily probability of malaria infection at each time step *t*, denoted *p*_*m*_(*t*) is calculated based on whether it is malaria season, interventions applied, and schistosomiasis infection status. *p*_*m*_(*t*) is calculated from the estimated number of infective bites per person per day, denoted *a*, which is given by the equation

$$a(t)=AEIR\left\{\frac{S}{D_{s}}M(t)+\frac{1-S}{D_{o}}[1-M(t)]\right\}[1-f_{net}(t)][1-f_{spray}(t)]f_{schisto}(t)$$ (1)

where *AEIR* is the annual entomological inoculation rate, in units of infective bites per person per year (see Parameters section and Table 3 below for discussion of user-selected *AEIR* value); *S* is the proportion of the *AEIR* over a single calendar year that occurs during peak malaria season; *D*_*s*_ is the duration of peak malaria season, in days; *D*_*o*_ is the duration of non-peak malaria season, in days (i.e., the rest of the year); *M*(*t*) is 1 if *t* represents a time step during peak malaria season, and 0 otherwise; *f*_*net*_(*t*) is the percent reduction in *AEIR* from the use of insecticide-treated bed nets (ITN) if distributed on or before time step *t*, and 0 otherwise; *f*_*spray*_(*t*) is the percent reduction in *AEIR* from the use of indoor residual spraying (IRS) if distributed on or before time step *t*, and 0 otherwise; and *f*_*schisto*_(*t*) is the increase in *AEIR* from being infected with schistosomiasis, equal to 1.85 representing an 85% increase from baseline malaria susceptibility if an individual has schistosomiasis during time step *t*, and 0 otherwise. In seasonal transmission regions (specified by the user), seasonal changes in *a* are represented directly in Eq 1 with *S* set to 0.8 for malaria season, thus allocating AEIR as 4-fold higher during peak transmission compared to off-peak transmission.

**Table 3:** User-assigned parameters for malaria transmission and interventions.

**Malaria: Transmission Pattern and Rate**			
Parameter name	Description	Default value	Possible values
Seasonality	Malaria transmission pattern: “Seasonal” or “Continuous”	Seasonal	“Seasonal” or “Continuous”
Peak Transmission Month(s)	Months of the year that are peak malaria transmission season	October through February	Any selection of calendar months including at least 1 month and not more than 11 months
Baseline infectious bites per year, AEIR (Annual entomological inoculation rate)	Annual entomological inoculation rate (# infective mosquito bites per person per year)	250	20 (low), 100 (medium), 250 (high) [15,16]
**Malaria: Interventions**			
Parameter name	Description	Default value	Possible values
Indoor Residual Spraying (IRS) Target % Coverage	Target percent coverage of IRS distribution	80%	0%, 30%, 50%, 80%, 100%
Indoor Residual Spraying (IRS) Current Distribution Month	Month IRS is distributed (for non-integrated/status quo treatment)	January	Any calendar month (e.g., “January”) or N/A if not chosen
Insecticide-treated Bed Nets (ITN) Target % Coverage	Target percent coverage of ITN distribution	80%	0%, 30%, 50%, 80%, 100%
Insecticide-treated Bed Nets (ITN) Current Distribution Month	Month ITN is distributed (for non-integrated)	November	Any calendar month (e.g., “November”) or N/A if not chosen

The daily probability of malaria infection *p*_*m*_(*t*) is then given by the equation

$$p_{m}(t)=1-\exp\left[-a(t)\right]$$ (2)

Each new malaria infection is assigned to one of four malaria infection states based on access to artemisinin-based combination therapy (ACT) treatment and the intrinsic likelihood that malaria infection causes symptoms:[15,16]

1. treated symptomatic malaria,
2. untreated symptomatic malaria,
3. untreated patent asymptomatic malaria, and
4. untreated subpatent asymptomatic malaria

Malaria symptoms are modeled by the probability $p_{symp}^{m}$ for *D*_*malaria*_ days. The model assumes those with symptomatic malaria seek and have access to artemisinin-based combination therapy (ACT) with probability $p_{treated}^{m}$ and become immune for *D*_*protect*_ days, with symptoms cleared after *D*_*malaria*_ days. Symptomatic malaria cases without access to ACT progress from the symptomatic phase to patent asymptomatic malaria lasting $D_{malaria}^{p}$ days and a phase of subpatent asymptomatic malaria illness lasting $D_{malaria}^{sp}$ days. People with asymptomatic malaria who do not seek treatment directly start a phase of patent and then subpatent asymptomatic malaria before clearing infection. Runs of the model in the absence of any interventions recapitulated aspects of previous modeling of schistosomiasis-malaria disease interactions, including the trend for schistosomiasis to increase malaria cases more in high malaria transmission and low ACT access settings (S1 Fig and S2 Fig). Each of these time-dependent transitions between malaria disease states are implemented at the person-level using timers that step with each day of the model. Malaria re-infections are handled in the same manner as new infections.

### Parameters

The model is populated with a series of fixed parameters chosen based on literature review to reflect countries where malaria and schistosomiasis are co-endemic, we selected Uganda, and additional user-specified parameter that represent conditions in their local community. Where data were not available from Uganda, the best available data from other co-endemic countries were used. For user selections, default values were also developed and the rationale for each of these parameters is outlined below.

#### Design of user-specified parameters

The user assigns model parameters from the user interface including population demographics, schistosomiasis prevalence, malaria transmission level and seasonality, and timing and coverage of schistosomiasis and malaria control measures (Table 2). These parameters were specifically chosen to reflect those data immediately available to in-country public health practitioners and include important factors in the design of disease control measures.

Schistosomiasis interventions often selectively target school-aged children (ages 5–15).[19] Therefore, demographic information is used to align MDA simulation with the approach in use on the ground. The model simulates populations with the demographic breakdown selected by the user and applies PZQ treatment to one or more age groups (under 5, 5–15, and 16+) since local MDA campaigns may target either the entire community or focus on school-aged children. National level demographics from our case country, Uganda, were chosen as the default conditions for the proof of concept effort[20] and can be modified by the user to reflect their community.

The efficacy of PZQ MDA depends upon schistosomiasis prevalence, coverage achieved during drug administrations, and the timing of the MDA program (whether MDA has been completed before peak malaria transmission). The default schistosomiasis prevalence is set at 45%, based on reports of medium and high intensity infections in the heavily endemic regions bordering Lake Victoria[21–24]; this parameter can also be customized by the user. The MDA default coverage is set at 80%, based on targets that communities have achieved with either school-based MDA or community-wide administration programs.[25] The MDA distribution month is community-specific and selected by the user to reflect the timing of current MDA programs.

Malaria seasonality and transmission rate are user-defined parameters (Table 3). Malaria transmission rates are defined as the daily infection probability based on the as infectious bites per year (ibpy): low (20 ibpy), medium (100 ibpy), and high (250 ibpy),[15,16] also known as the annual entomological inoculation rate or AEIR. The peak transmission month(s) have an increased infection probability approximated by a four-fold increase in transmission rates during peak transmission months. That is, malaria peak seasonal transmission, *S*, is equal to the proportion of the AEIR over a single calendar year that occurs within peak malaria season where seasonal variation is assumed to be *S* = 0.8 based on estimates of seasonal changes in EIR measured in Uganda[16,26] and is consistent with the differential transmission rates between regions with seven or more months of favorable climate for malaria transmission compared to regions with six months or fewer.[27] The baseline AEIR determines seasonal and continuous (year-round) infection probabilities.

Users input parameters for defining the current timing and coverage for IRS and distribution of ITNs. Interventions (access to IRS and/or ITN) are assigned randomly in the model, without respect to age, to simulate target coverage rates specified by the user as what the local community expects it can achieve as part of a malaria control program (e.g., 80% IRS coverage equates to 0.8 individual probability of receiving protection from IRS). Modeled interventions are assumed to provide protection throughout the modeling period.

Default simulation size of 2,000 people (*N*_*people*_) and baseline stabilization of 730 days ($N_{days}^{burn}$) are used in all example simulations presented here (Table 4). Both the population size and the burn in period were selected based on empirical testing. A 730 day burn in was sufficient to stabilize baseline malaria prevalence. Each simulation lasts 365 days (*N*_*days*_) after the initial stabilization to evaluate predicted disease prevalence in the first year following integration of disease control programs.

**Table 4:** Static model parameters for model duration and population size.

Parameter name	Description	Value	Source
*N*_*people*_	The number of people being simulated	2,000	Parametrically determined
*N*_*days*_	The number of days being simulated	365	
$N_{days}^{burn}$	The minimum number of days the model is equilibrated prior to the 365 day simulation	730	

Parameters used to model the protections afforded by control measures are summarized in Table 5. These factors are used to adjust AEIR for the protective interventions (IRS and ITN) and increased susceptibility to malaria infection for schistosomiasis-infected individuals. These factors are combined in calculating the probability of a susceptible person contracting a new malaria infection (*p*_*m*_(*t*)) using Eqs 1 and 2 (see above). Malaria infection probability for individuals is adjusted based on IRS and ITN efficacy estimates found in studies conducted in Kenya and a meta-analysis of protection provided by malaria interventions in endemic countries.[28,29] Co-infection with schistosomiasis increases risk to malaria by 1.85.[15]

**Table 5:** Model parameters that determine malaria infection probability (*p*_*m*_(*t*)).

Parameter name	Description	Value	Source
*f*_*net*_(t)	Percent reduction in *AEIR* due to use of insecticide-treated bed nets	53% (0.53) if nets were distributed on or before time *t*, and 0 otherwise	Efficacy from in-country study in Kenya[28]
*f*_*spray*_(*t*)	Percent reduction in *AEIR* due to use of indoor residual spraying	65% (0.65) if sprays were distributed on or before time *t*, and 0 otherwise	Average of results from in-country and meta-analysis studies[28,29]
*f*_*schisto*_(*t*)	Percent increase in *AEIR* due to being infected with schistosomiasis	85% (1.85) if individual has schistosomiasis at time *t*, and 0 otherwise	From established model of schistosomiasis-malaria disease interactions[15]

Malaria treatment is modeled for symptomatic cases ($p_{symp}^{m}$) based on access to ACT treatment, as determined using an estimate of ACT availability in malaria endemic countries.[15,16] Disease parameters include the duration of symptomatic malaria *D*_*malaria*_ and the duration of temporary protection from malaria infection following ACT treatment *D*_*protect*_ (Table 6). The duration of asymptomatic malaria disease states ($D_{malaria}^{p}$ and $D_{malaria}^{sp}$) track asymptomatic malaria cases and include these cases in population malaria prevalence.[15,16] Symptomatic malaria cases receive ACT treatment 76% of the time using a maximal ACT treatment access estimate for Uganda.[17]

**Table 6:** Parameters determining symptomatic malaria cases and access to treatment.

Parameter name	Description	Value	Source
$p_{symp}^{m}$	Probability that a person infected with malaria is symptomatic	0.72	From established malaria disease models[15,16]
$p_{treated}^{m}$	Probability that a person infected with symptomatic malaria is treated with ACT	0.76	Estimated access to ACT treatment in Uganda[30]
*D*_*protect*_	Duration of malaria protection following ACT treatment	20 days	From established malaria disease models[15,16]
*D*_*malaria*_	Symptomatic malaria duration	5 days	
$D_{malaria}^{p}$	Patent asymptomatic malaria duration	180 days	
$D_{malaria}^{sp}$	Sub-patent asymptomatic malaria duration	180 days	

### User interface

A graphical user interface (GUI) provides access to the model for practitioners through a web application (HTML5, JavaScript, and CSS3). The web application uses NodeJS, built on a Python-shell NodeJS package, to interface with the back-end model code written in Python and accessible from an internet-connected computer. The user interface is designed to be intuitive, to use plain language understandable to public health practitioners as tested with the end user community, and to guide users through inputs used to align the model run with disease transmission patterns and the current state and constraints of existing disease control measures for schistosomiasis and malaria. Default selections are built into the interface as a proof-of-concept for application of the model in co-endemic countries with high rates of co-infection. The user interface also provides a results page with a set of decision-focused graphics that apply the results of the back-end model specifically to support evaluation of the predicted benefits, if any, of integration of schistosomiasis and malaria control programs and support the decision to integrate or not.

## Results

By combining literature-based parameter selection with user and decision-focused design, we found that a web-based decision support tool is well-suited to adapt core approaches to disease modeling, including schistosomiasis-malaria disease interactions capturing increased malaria susceptibility for individuals infected with schistosomiasis, for use by public health practitioners. User inputs, made through the web-based interface, populate the model with data to approximate conditions in the local community. Results of parallel simulations (non-integrated and integrated) are summarized in decision-focused formats designed to provide the information required for local public health practitioners and policy-makers to evaluate the benefits of an integrated control program for schistosomiasis and malaria. The decision support tool is designed to provide the results most relevant to decision makers: prevalence is compared between conditions in which control measures are integrated or not (the latter being the assumed baseline condition.) This method provides communities a practical comparative analysis to assess the utility of integration based on local disease conditions.

### Modeling integrated and non-integrated interventions

**Fig 2:**
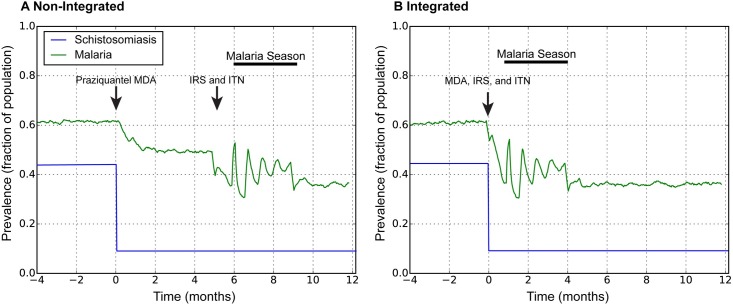
Example modeling results for a very high transmission region with seasonal malaria transmission. Graphs shows prevalence of malaria (green) and schistosomiasis (blue) at baseline (prior to time zero) and in the months following sequential, non-integrated (A) or integrated interventions (B). Arrows mark the timing of malaria and schistosomiasis control measure distribution.

The decision support tool allows the end user to compare the prevalence rates of both diseases in their community under non-integrated and integrated disease control programs by modeling the protective effects of differently timed disease control interventions (Fig 2). Simulations (integrated and non-integrated) are run for a minimum of two years to provide a stable baseline estimate of prevalence (-4–0 months are from the initial baseline period). In the non-integrated simulation, the first intervention is delivered at time 0, in this example distribution of PZQ MDA to all age groups with 80% population coverage. PZQ distribution is instantaneous in the model and, in this example, immediately reduced the simulated prevalence of schistosomiasis (Fig 2, blue), and produced an indirect benefit by reducing malaria prevalence (Fig 2, green). Malaria prevalence was further reduced in the non-integrated simulation by the distribution of ITN and application of IRS despite the seasonal increase in malaria transmission that occurs between the 6^th^ and 9^th^ month of the simulation. In the integrated simulation, malaria control measures and PZQ MDA occur simultaneously (Fig 2, Time 0). The example region shown here has a seasonal malaria transmission pattern; PZQ, IRS, and ITN are applied one month prior to the start of malaria season. The integrated approach (Fig 2B) had a lower average prevalence for the post-intervention period relative to the non-integrated approach (Fig 2A) (43% integrated vs. 50% non-integrated) indicating that the integrated control program was more effective in reducing malaria prevalence under these conditions, as produced by the Python computational model (the raw outputs shown are plotted directly from the back-end model).

### Data-driven decision support for local public health officials

The complex results shown in Fig 2 provide data, but the relevant information is not immediately obvious, especially for end users or decision makers with limited time or without expertise in disease modeling. The data produced by the model can be more effectively communicated using a web-based decision support tool, as described here. In this tool, the end user supplies readily available information about their community (i.e., parametric data) to define local conditions, and results are returned as a recommendation for or against integration, with context and practical application guidance.

**Fig 3:**
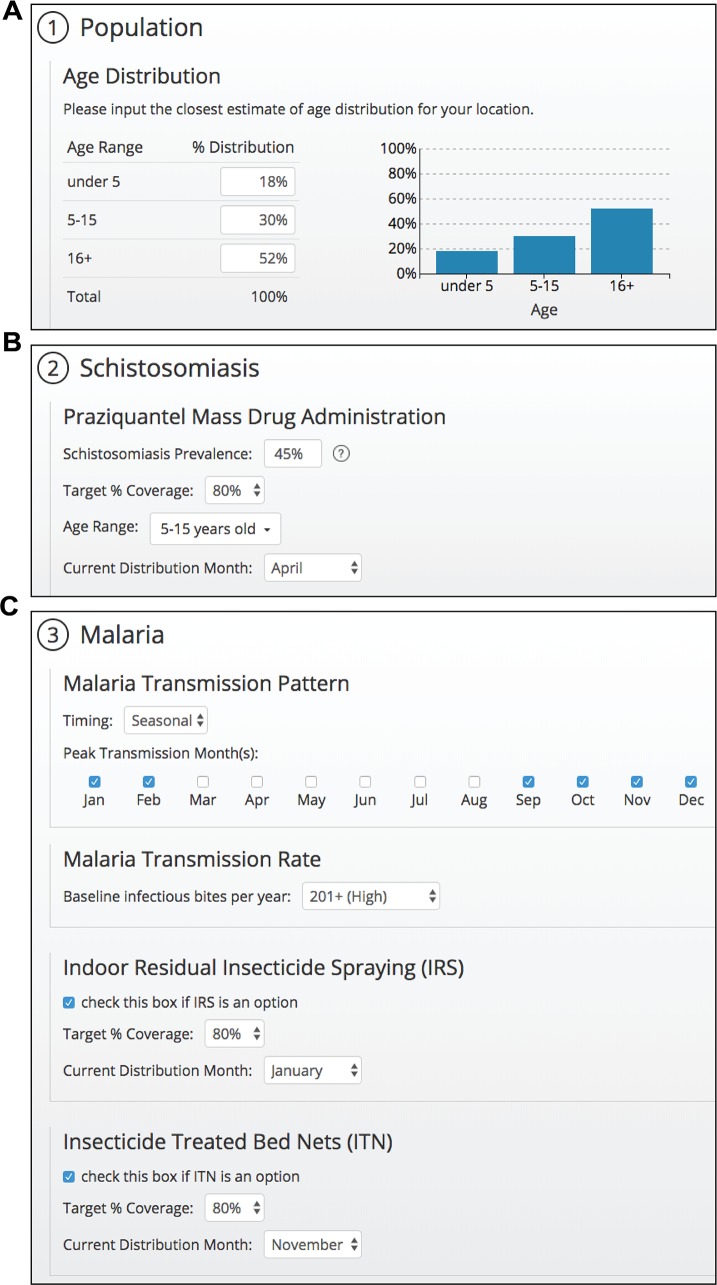
Design of a user selection page for population breakdown and disease control programs. (A) Demographics from Uganda at the national level were chosen as the default conditions for the proof of concept effort and can be modified by the user to reflect their community. (B) Users select the target treatment coverage for praziquantel MDA, select one or more age groups from the dropdown menu, and select a single month when MDA interventions are completed. (C) Malaria transmission pattern is specified as seasonal or continuous (year-round) and peak transmission months are selected for seasonal transmission (top). Malaria transmission rate is set as high, medium, or low and users are provided with the equivalent rate of infectious bites per year (middle). Users also specify the timing and target coverage for existing malaria control programs (bottom).

The benefits of integration in a region depend upon schistosomiasis prevalence, access to PZQ MDA, and the types and coverage of malaria control measures, data on which should be collected by those implementing public health control measures locally or regionally[31,32], and are supplied by users of the decision support tool and applied as parameters for the model (Fig 3). As shown in Fig 3, the decision support tool user interface guides users through a step-by-step workflow to choose inputs. Default demographic information, drawn from national data for Uganda,[20] was used in all simulations presented here, but the population, schistosomiasis, and malaria selections can each be customized to match the user’s community.

Transmission rate and seasonality have significant impacts on the efficacy of malaria inventions[18,26,33] and, by extension, are expected to affect the efficacy of an integrated control program for schistosomiasis and malaria. As with key schistosomiasis parameters, malaria seasonality and transmission rate, as well as current local or regional implementation of IRS and distribution of ITNs are user-defined inputs in the tool (Fig 3C).

**Fig 4:**
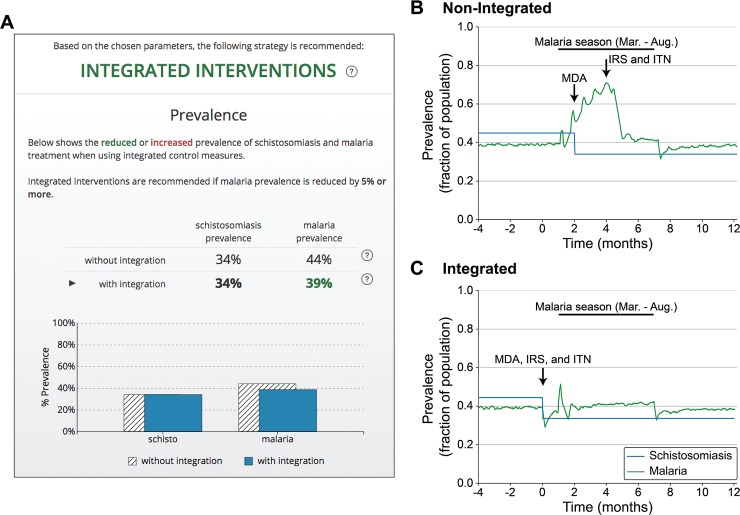
Example of decision-focused summary and modeling results. (A) Results page summary for a region where an integrated approach is recommended. Results page summarizes estimated difference in the prevalence between intervention strategies in tabular and graphical formats. (B-C) Model results for malaria prevalence (green) and schistosomiasis prevalence (blue) used as the basis for the comparison in (A) by averaging the prevalence across the terminal year of each model run.

**Fig 5:**
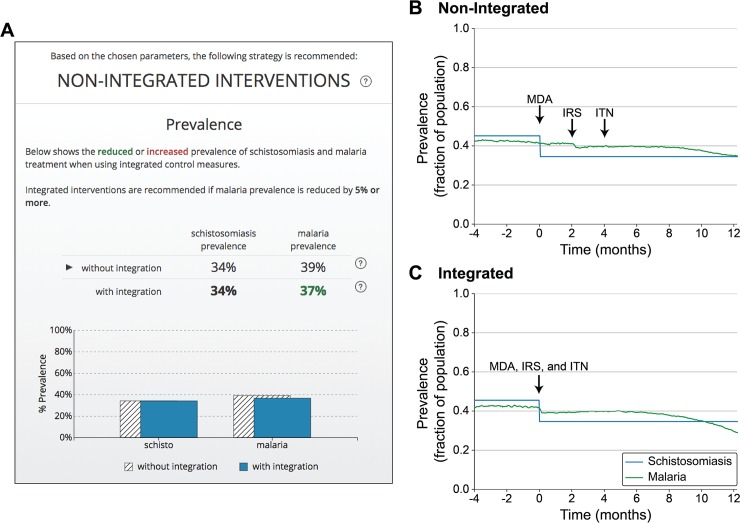
Example of decision-focused summary and modeling results. (A) Results page summary for a region where a non-integrated approach is recommended. Results page summarizes estimated difference in the prevalence between intervention strategies in tabular and graphical formats. (B-C) Model results for malaria prevalence (green) and schistosomiasis prevalence (blue) used as the basis for the comparison in (A) by averaging the prevalence across the terminal year of each model run.

The results produced by the model are presented in the decision support tool on a single page specifically designed to focus the end user on the recommendation produced by the model, context for that recommendation, and practical implementation guidance for either integrated or non-integrated control programs (Fig 4A, Fig 5A). As described in additional detail for two example regions below, model runs are summarized in graphics that support he rapid comparison of the relative benefit of integrated and non-integrated programs.

For a notional community with high malarial transmission and a six-month malaria season, the results presented by the decision support tool suggest integration of schistosomiasis and malaria disease control programs based on a reduction of malaria prevalence by 5% compared to the current strategy (PZQ MDA distributed early in malaria season with IRS and ITN interventions mid-season) (see Fig 4A–4C). Initial schistosomiasis prevalence was modeled at of 45% with subsequent reduction by school-based PZQ MDA of children aged 5–15, either during malaria season for the non-integrated approach (Fig 4B) or prior to malaria season in the integrated approach (Fig 4C). This treatment indirectly reduced seasonal malaria transmission and yielded a corresponding reduction in peak and average malaria prevalence of 5% (44% malaria prevalence without integration vs. 39% with integration across the year-long simulation). This degree of reduction meets the 5% threshold for applying a recommendation of integrated treatment in the decision support tool. Using a specific threshold value for recommending integrated interventions, and providing this information to the user, the tool provides clear guidance for decision makers. The results page explains that “Integrated interventions are recommended if malaria prevalence is reduced by 5% or more” (Fig 4A, Fig 5A). Increasing the model run duration produced similar results with respect to the predicted benefits of integration across different regions, including for the example regions from Figs 4 and 5 (3-year simulation: 41% malaria prevalence without integration vs. 38% with integration for the region with integration recommended; 32% malaria prevalence without integration vs. 31% for the region where integration not recommended). Importantly, future testing is planned to evaluate the proof of concept decision support tool with community-level public health decision makers, including evaluating the selection of 5% malaria prevalence reduction as the cutoff for recommending integration of control measures and the use of a first-year window to evaluate prevalence (see Discussion).

Integration of schistosomiasis and malaria disease control programs does not provide large malaria prevalence reductions for all regions; the decision support tool recommends retaining a default non-integrated control program in cases where minimal reductions in malaria prevalence are predicted. For example, as shown in Fig 5A–5C, results in a low malaria transmission region with a continuous (year-round) transmission pattern suggest that, when schistosomiasis prevalence and MDA for school-aged children was modeled the same as for a high, seasonal transmission region, integrating disease control programs to align MDA, IRS application, and ITN distribution (Fig 5C) provided minimal benefit for peak malaria prevalence compared to delivering the interventions sequentially (Fig 5B). Integration also provided only a small benefit for average annual malaria prevalence compared with the current approach (39% malaria prevalence for non-integrated vs. 37% for integrated). As shown in this case, for conditions under which the community is recommended to preserve the current approach, the tool provides a summary of the estimated prevalence, as it does for the integrated method, to provide context for the result and corresponding decision (Fig 5A).

**Fig 6:**
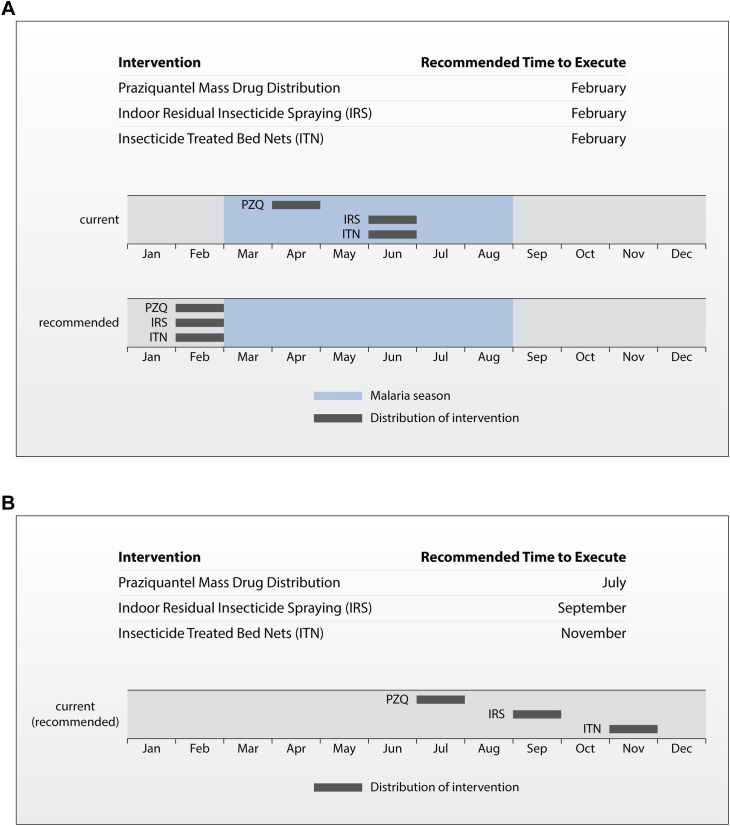
Example distribution timeline from the results page. Summary table and timeline of the current (non-integrated) and recommended distribution times for control measures. (A) This example corresponds to a region were integration of control programs is predicted to reduce malaria prevalence compared to the current distribution strategy. (B) Summary table and timeline for a region where integration is not recommended based on modeling results. Distribution is matched to the current intervention strategy provided by the user.

To use the tool, decision makers are required to input detailed information about the modeled disease and current control method implementation in their community. Using this information, the decision support results section also includes an implementation strategy for control methods under either integrated or non-integrated conditions, as recommended based on the modeling results (Fig 6). The distribution strategy outlines a timeline that summarizes the application time for each intervention with respect to the current distribution strategy and, if applicable, malaria season. The recommended distribution timeline for the high, seasonal transmission region considered in Fig 4 aligns PZQ MDA, IRS, and ITN to occur in February to precede the malaria season. As shown by comparison to the current intervention timeline, this requires moving PZQ MDA two months earlier in the year and IRS and ITN interventions four months earlier. For the second region, where integration was not recommended, the non-integrated distribution timeline shows the current strategy defined by the user in the inputs. Summarizing the execution timing for each disease control measure, and providing context for how the recommended strategy compares to the current approach, directly supports public health decision makers to act on the decision support tool results, communicate the results to stakeholders, and plan for implementing integration.

## Discussion

Our model and decision-support tool demonstrates the utility of a user-friendly yet evidence-based approach to disease integration, allowing decision-makers access to the best available scientific evidence with which to design interventions tailored to their particular setting. For example, timing the delivery of malaria control measures to coincide with PZQ MDA affords the opportunity to combine control programs with limited resources to better-resourced programs and maximizes the impact of both programs on disease prevalence. However, the outcomes of our decision-support tool also describe conditions under which integration may not have significant epidemiological benefits; given the significant investments of human and financial capital required to integrate disease control programs, our work highlights the importance of assessing the specific benefits of integration prior to making decisions regarding near or long-term programmatic investments.

The people who make the practical decisions about how to implement public health interventions in their communities, such as integrating control programs, have a broad and challenging task, particularly in communities where disease is endemic and funds are limited. The organizations involved in helping support or fund disease control programs may not coordinate closely and are often focused on one or two diseases, frequently determined by funding sources or restrictions as opposed to on-the-ground conditions. To improve the ability of both groups to apply the best available modeling and academic analysis in implementing public health interventions despite limited time, funding, and, potentially, disease-specific expertise, we describe here a generalizable method to adapt established computational disease models to support and inform practical decisions about implementing public health control measures in the communities most directly affected.

By summarizing complex disease model outputs as a straightforward benefit comparison, in language and terminology familiar to disease control officers, our prototype tool provides the effective translation from academic research to support evidence-based decision making at national, regional, and local scales. A web-based tool provides an interface for non-experts to evaluate potential intervention strategies, as compared to currently implemented disease control programs, based on their own local information about disease prevalence and transmission patterns. Placing the best available predictive data in the hands of in-country public health practitioners enhances their ability to make difficult decisions about public health investments in resource-constrained environments and communicate these results to others.

The proof-of-principle decision support tool, as described here, evaluates the potential of an integrated schistosomiasis and malaria disease control program to reduce malaria prevalence for a specific community to directly support local decisions about integrating control programs for the two diseases and is, therefore, specifically targeted to communities in which schistosomiasis and malaria are co-endemic. Like many co-endemic diseases, the interaction between schistosomiasis and malaria has complicated epidemiological and intra-host disease interactions that directly influence the complexity of control strategies. The focus of this effort was to test the adaptation of a complex computational disease model in to a tool useful for local and regional decision-making. The tool incorporates approaches to schistosomiasis and malaria disease modeling established in the literature[16] for testing disease control interventions. In addition, we incorporated a more recently-developed approach to modeling schistosomiasis-malaria disease interactions whereby for schistosomiasis-infected individuals have increased susceptibility for malaria,[15] though our proof of concept method includes simplifications from previously-published epidemiological models, including a static representation of schistosomiasis disease prevalence rather than a full adaptation of a dynamic model of schistosomiasis transmission. Future work to expand upon this proof-of-concept decision support tool will include user and field testing to target any expansions of the model to the parameters identified as most sensitive to local conditions and critical to decisions in the field. By building a targeted decision support tool that is readily understood by public health practitioners and focused on practical decision making on the ground, we have a strong basis for future refinement of the approach for control of schistosomiasis and malaria or other co-endemic diseases.

Future model development to incorporate additional vector and epidemiological dynamics, and particularly schistosomiasis re-infection, will further refine the representation of disease transmission in endemic settings. This can further include additional population-level dynamics, such as the potential for praziquantel MDA to reduce transmission and protect even those who were not directly treated.[34] Indeed, advances continue in development of research models for schistosomiasis, malaria, and co-infection disease dynamics as well as approaches to model prevention and treatment interventions. Recent work demonstrates, for example, that controlling schistosomiasis infections can contribute to population-level control of malaria independent of any direct increase in malaria susceptibility from schistosomiasis infection within individual hosts.[35] Using the method described here for translating the results of such research into practical applications provides a powerful tool to apply the most up-to-date research methods are applied directly to the challenges faced by those tasked with implementing disease control measures on-the ground.

As with all modeling, low data availability can limit the quality of parameter values used in assessing the benefits of integrated disease programs. However, the flexible decision support platform described here is designed to incorporate more refined data as it becomes available, including at the local level. For example, while intervention programs may specifically promote access to ITNs for the most vulnerable populations, such as pregnant women and young children, it is much more difficult to determine the utilization of these interventions–for example, whether the children are actually sleeping under the net. The academic literature cites similar uncertainties related to drug availability and uptake of appropriate first line antimalarial medication. We anticipate that further research, and particularly in-country data validation and testing of the decision support tool, will provide additional information on the true extent of age- and population-specific usage of ITNs and other interventions. As additional information on these parameters is gathered, revised, age-specific values for access to control measures and treatment can be readily incorporated into the modeling framework using the same user-supplied demographic information as is already in place for the schistosomiasis MDA intervention. The flexibility of this decision support framework provides the ability to continuously update the parameters and ensure the best available data, including empirical data from the users themselves, is incorporated to support decision-making.

Particularly when communicating the result of complex analysis to non-expert users, it is important to limit quantitative information, while focusing on that information specifically relevant to the decision; providing the results in plain language and limiting technical terminology; and focusing on the practical result of the guidance provided[36–38]. These principles were specifically considered and applied to the results presented in the user interface. Future efforts will focus on expanding user testing to confirm that the methods applied were effective and both the results and technical content were sufficiently described for use by the intended end user.

We performed initial validation of the value of the tool to end-users by soliciting feedback from partners in schistosomiasis and malaria endemic countries. Responses from partners from Mali, Uganda, and Yemen, were strongly positive, confirming the utility of this type of simple yet powerful tool, driven by data, to provide recommendations *a priori* during decision-making. The integration of disease programs is a major upheaval in many communities and requires significant capital in terms of human, financial, and other resources, so being able to ascertain the expected benefits–if any–is an important step forward. The feedback also identified opportunities to expand the model to incorporate both the additional schistosomiasis parameters as described above, but also resource management in the implementation of control interventions. Specifically, our partners noted that the alignment of resource allocation would be an important factor to consider when deciding whether to integrate across different diseases; indeed, distribution and logistical issues can be a major constraint to intervention delivery, particularly when decisions are made nationally but require implementation at the local level. To address these realities, a module that examines cost-effectiveness of interventions in the context of resource allocation and distribution will be an important addition for expansion of the decision support tool. Future work will include expanding the tool to include such analysis to predict logistical or distribution bottlenecks during implementation, and the results used for advocacy to the national government or donors for more effective resource allocation.

We present results of an approach to apply academic disease modeling research to practical public health implementation efforts through development of a user-friendly decision support tool. This evidence-based approach to integrated of control programs has implications beyond NTDs and even the Big Three. Conceptually, this approach could be used for integration of any diseases or health interventions–for example, to examine opportunities for aligning water, sanitation and hygiene (WASH) interventions with those for NTDs or diarrheal diseases, or evaluating whether maternal and child health programs could be leveraged for other aspects of disease control. By emphasizing consideration of horizontal public health programs, potential constraints from vertical programs can be avoided and support interventions that contribute to a more resilient and effective overall health system. By combining variables and outcomes for the target programs at the outset, this approach might also be applied to overcoming some of the challenges associated with a lack of shared indicators for evaluation or cohesive overall strategy that has been observed in other attempts to integrate across sectors[39]. Finally, in this era of increasingly constrained resources for global health, our approach provides an opportunity to link epidemiological evidence with intervention costs, to optimize delivery of health services and effectiveness of control programming.

## Supporting information

S1 Fig Schistosomiasis-malaria disease interaction increases malaria cases in a transmission-dependent manner. Number of symptomatic malaria cases attributed to the modeled increase in malaria susceptibility for schistosomiasis-infected individuals. Model results were compared with and without an 85% increase in malaria susceptibility to determine the effects of schistosomiasis on the number of symptomatic malaria episodes. All disease control measures were excluded, ACT coverage was set to 60%, and AEIR was varied from 10–250 ibpy.
(TIF)

S2 Fig Schistosomiasis-malaria disease interaction has decreased effects as ACT access increases. Number of symptomatic malaria cases attributed to the modeled increase in malaria susceptibility for schistosomiasis-infected individuals. Model results were compared with and without an 85% increase in malaria susceptibility to determine the effects of schistosomiasis on the number of symptomatic malaria episodes. All disease control measures were excluded, AEIR was fixed at 100 ibpy, and ACT coverage was varied from 20–80%.
(TIF)
