# Supplementary figures and images for: Decision support for evidence-based integration of disease control: A proof of concept for malaria and schistosomiasis
Source: PLoS Negl Trop Dis. 2018 Apr 12;12(4):e0006328. doi: 10.1371/journal.pntd.0006328 (PMC5896906; doi:10.1371/journal.pntd.0006328)

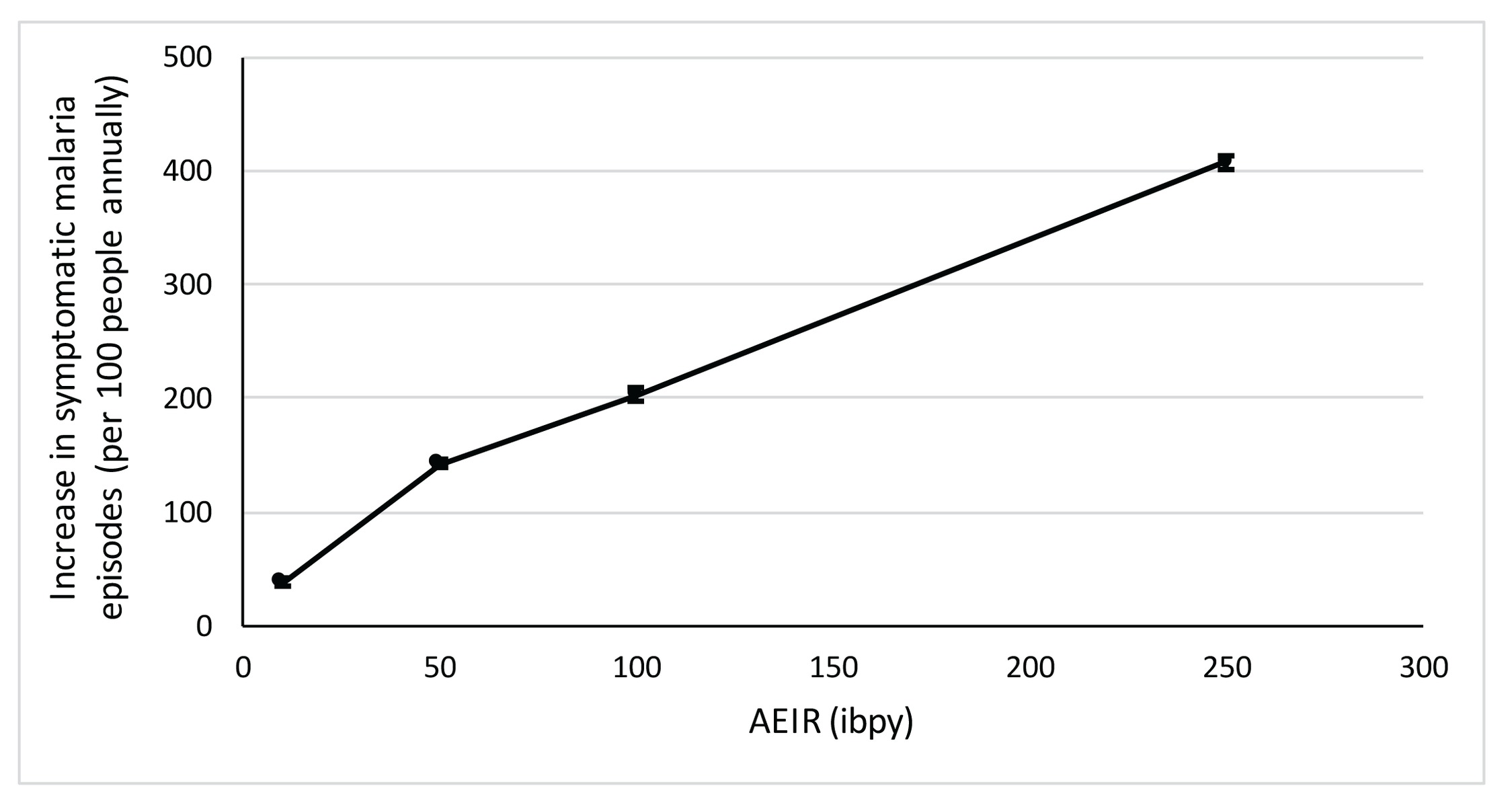

Supplement: S1 Fig — Number of symptomatic malaria cases attributed to the modeled increase in malaria susceptibility for schistosomiasis-infected individuals. Model results were compared with and without an 85% increase in malaria susceptibility to determine the effects of schistosomiasis on the number of symptomatic malaria episodes. All disease control measures were excluded, ACT coverage was set to 60%, and AEIR was varied from 10–250 ibpy. (TIF) [file pntd.0006328.s001.tif]

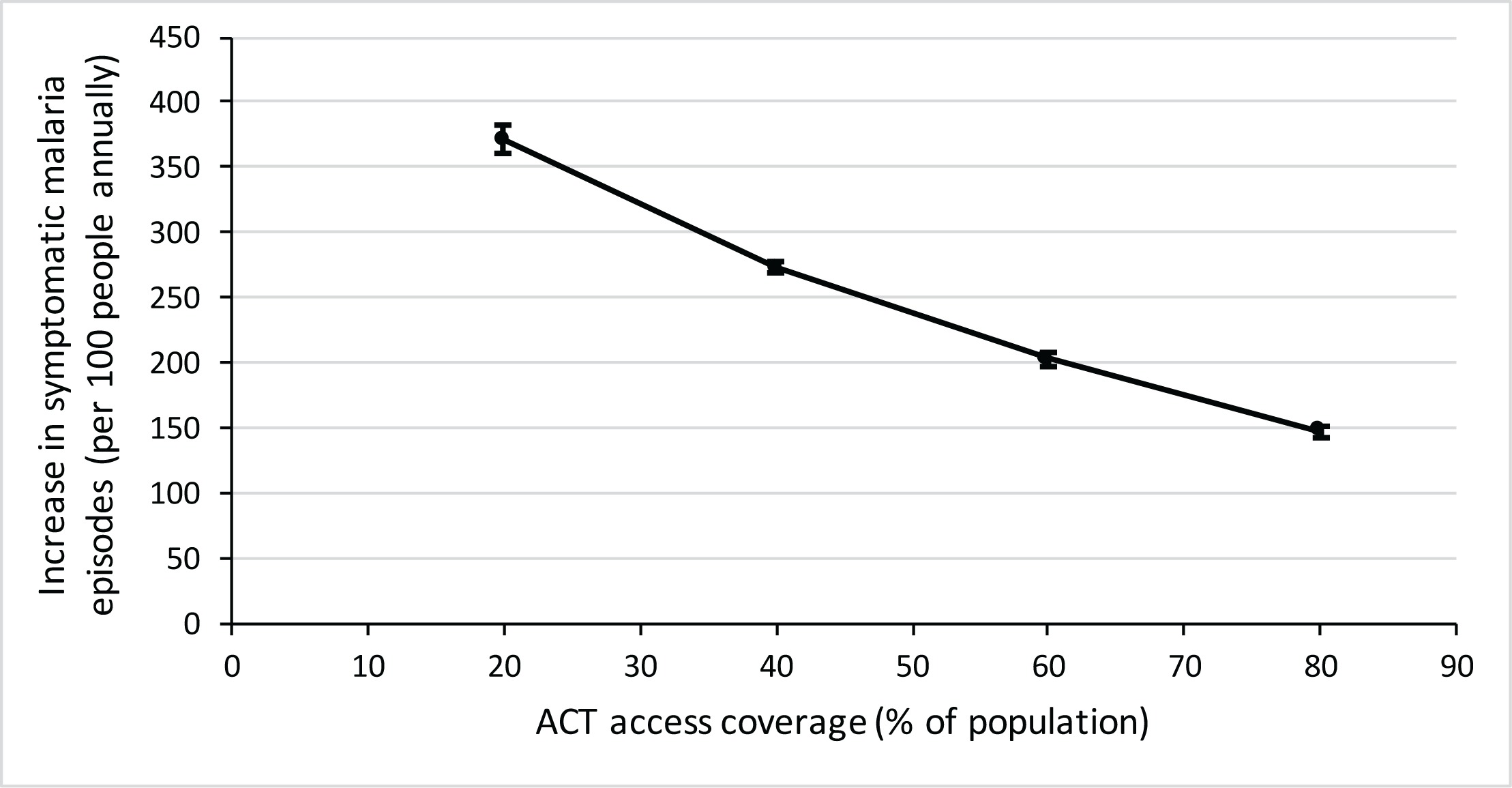

Supplement: S2 Fig — Number of symptomatic malaria cases attributed to the modeled increase in malaria susceptibility for schistosomiasis-infected individuals. Model results were compared with and without an 85% increase in malaria susceptibility to determine the effects of schistosomiasis on the number of symptomatic malaria episodes. All disease control measures were excluded, AEIR was fixed at 100 ibpy, and ACT coverage was varied from 20–80%. (TIF) [file pntd.0006328.s002.tif]
